# Comprehensive Analysis of Cyclin Family Gene Expression in Colon Cancer

**DOI:** 10.3389/fonc.2021.674394

**Published:** 2021-04-29

**Authors:** Jieling Li, Liyuan Zhou, Ying Liu, Lingzhi Yang, Dayi Jiang, Kuan Li, Shouxia Xie, Xiao Wang, Shaoxiang Wang

**Affiliations:** ^1^ School of Pharmaceutical Sciences, Shenzhen University Health Science Center, Shenzhen, China; ^2^ Department of Pharmacy, Shenzhen People’s Hospital (The Second Clinical Medical College, Jinan University, The First Affiliated Hospital, Southern University of Science and Technology), Shenzhen, China

**Keywords:** cyclin, colon cancer, gene expression, diagnosis, prognosis, biomarker

## Abstract

Colon cancer is a common malignancy of the digestive tract with high morbidity and mortality. There is an urgent need to identify effective biomarkers for the early diagnosis of colon cancer and to prolong patient survival. Cyclins are a family of proteins that directly participate in the cell cycle and are associated with many types of tumors, but the role and regulatory mechanism of most cyclin family members in colon cancer remain unclear. Here, we provide a systematic and comprehensive study of cyclin family gene expression and their potential roles in colon cancer. Pan-cancer analysis revealed that cyclin genes were most differentially expressed in colon adenocarcinoma. Among the four datasets of colon cancer from The Cancer Genome Atlas and the Gene Expression Omnibus, six cyclin genes (*CCNA2*, *CCNB1*, *CCND1*, *CCNE1*, *CCNF*, and *CCNJL*) were differentially expressed between normal and tumor tissues. Four of them (*CCNA2*, *CCNB1*, *CCNE1*, and *CCNF*) were notably elevated in the early TNM stages and significantly correlated with overall survival. Meanwhile, the expression of *CCNA2* and *CCNB1* was positively correlated with tumor-killing immune cells, such as CD8+ T cells.The copy numbers of *CCNA2*, *CCNB1*, *CCND1*, *CCNE1*, and *CCNF* was positively related to gene expression. The methylation levels of *CCNB1* were lower in tumor tissues than in normal tissues and were negatively correlated with gene expression. The receiver operating characteristic curves indicated that the gene expression of 24 cyclins had higher predictive accuracy than the TNM stage. Pathway analysis showed that cyclin genes were tightly associated with apoptosis, the cell cycle, hormone ER, the RAS/MAPK pathway, mismatch repair, mTORC1 signaling, KRAS signaling, Akt, and TGFB in colon cancer. Weighted gene co-expression network analysis suggested that cyclin genes were closely linked to *CDK1*, *BIRC5*, *PLK1*, and *BCL2L12*. At the protein level, Cyclin A2 and Cyclin B1 were also expressed higher in colon adenocarcinoma tissues. In addition, cyclin genes were highly related to the drug sensitivity of some FDA-approved drugs, such as MEK and EGFR inhibitors, which might provide guidance for clinical treatment. In conclusion, cyclin genes are promising biomarkers for the diagnosis and prognosis of colon cancer.

## Introduction

As reported by the 2018 Global Cancer Statistics, colon cancer is the fourth most commonly diagnosed cancer and ranks fifth in terms of cancer mortality rates (1). There are effective therapeutic methods for patients with colon cancer, such as surgery, chemotherapy, and radiofrequency ablation (2). However, due to metastasis, the five-year survival rate of patients with stage I colon cancer is 92%, while that of patients with stage IV is only 11% (3). Unfortunately, approximately 20% of patients with colon cancer are diagnosed at stage IV every year (4). Therefore, the identification of ideal and efficient biomarkers to help diagnose patients at an early stage is urgently needed.

Cyclins are a group of proteins that regulate the cell division cycle by binding and activating cyclin-dependent kinases (CDKs) (5). The gene group of cyclins consists of 31 members according to the HUGO Gene Nomenclature Committee, an internationally recognized institute providing gene information (https://www.genenames.org) (6). The dysregulation of *CCND1* and *CCNE1* is linked with malignant cell transformation, and they are overexpressed in several types of tumors (7, 8). We investigated the expression patterns of cyclin family genes in different types of cancers using Gene Set Cancer Analysis (GSCA), a website that collects the cancer genomics data of 33 cancer types from The Cancer Genome Atlas (TCGA) database. We found that cyclin genes were most differentially expressed in colon adenocarcinoma (COAD). It was reported that *CCNB1* and *CCND1* were elevated in colon tumor tissues (9, 10). However, the relationships between many other cyclin family members and colon cancer remain largely unexplored.

This study aimed to provide a systematic and comprehensive study of cyclin family gene expression and illuminate their potential roles in colon cancer. We collected clinical information, gene expression profiles, and copy number data of patients with colon cancer from TCGA database and the Gene Expression Omnibus (GEO). First, we performed differential expression analysis to determine the differentially expressed cyclin genes between normal and tumor samples to identify possible diagnostic markers. We also investigated the copy number and DNA methylation of cyclin genes in COAD. Then the univariate Cox regression analysis was used to examine the correlation between patient overall survival and clinicopathological characteristics.The prognostic value of cyclin genes was evaluated by Kaplan-Meier survival curves, Cox proportional hazards model, and nomograms. Moreover, gene set enrichment analysis (GSEA) was applied to explore the cyclin-related pathways, which may shed light on the underlying molecular mechanisms of cyclin genes in colon cancer. A gene co-expression network was constructed by weighted gene co-expression network analysis (WGCNA) and Cytoscape to show the genes closely linked with cyclins. To further confirm the results of gene expression analysis, we analyzed the protein expression data of cyclins from the Human Protein Atlas (HPA) and CPTAC data portal using similar methods.

## Materials and Methods

### Sources and Types of the Data

The clinical data of patients with colon cancer were downloaded from TCGA database and three datasets (GSE39582, GSE41258, and GSE44076) of the GEO database (https://www.ncbi.nlm.nih.gov/geo). In total, 452 cases from TCGA, 566 from GSE39582, 169 from GSE41258, and 98 from GSE44076 were acquired. The gene expression profiles of normal and tumor tissues from patients with colon cancer were also extracted from the four datasets (41 normal samples and 456 tumor samples from TCGA, 19 normal samples and 566 tumor samples from GSE39582, 54 normal samples and 172 tumor samples from GSE41258, and 98 normal samples and 98 tumor samples from GSE44076). The copy number data of patients with COAD were obtained from TCGA (Data type: Masked Copy Number Segment). The immunohistochemical data of cyclin protein expression in COAD and normal tissues were collected from the HPA database (https://www.proteinatlas.org/). The protein levels of cyclins in COAD were downloaded from the CPTAC data portal (https://cptac-data-portal.georgetown.edu/; S037; Proteome of PNNL).

### Analysis of Cyclin Gene Expression

The gene expression of cyclin family members in different cancers was explored by GSCA (http://bioinfo.life.hust.edu.cn/GSCA). Fourteen cancers with paired samples were included in the analysis. FireBrowse (http://firebrowse.org/) was used to show the expression levels of the top six cyclin genes in normal and tumor tissues of different cancers from the results of GSCA.

Differential expression analysis was adopted with the edgeR (11) and limma (12) packages in R software (https://www.r-project.org/; v3.5.3) for TCGA and GEO data, respectively. Then, the differentially expressed genes (DEGs) of 24 cyclin family genes (four datasets had these in common) were selected and their expression patterns were presented with heatmaps. A Venn diagram was drawn to show the mutual DEGs among the four datasets. Boxplots of the mutual DEGs were also plotted by R.

Immunohistochemical images from the HPA were analyzed by Image-Pro Plus software (v6.0). The average density of protein expression was measured by the integrated optical density and the area. The bar plot of expression density was drawn by GraphPad Prism software (v6.0).

To construct a co-expression network, the WGCNA package (13) was employed to assess the correlation coefficients between the important cyclin genes and other DEGs using the expression profiles of tumor tissues in TCGA dataset. The top 30 genes most correlated with each cyclin gene were selected, and their network was drawn by Cytoscape (http://www.cytoscape.org/; v3.7.1).

### Relationships Between Cyclin Genes and Patient Survival

The correlation between overall survival and clinicopathological features of the patients was analyzed using univariate Cox regression analysis. Patients with a follow-up time shorter than 30 days were excluded. Finally, 421 cases from TCGA, 556 from GSE39582, and 167 from GSE41258 were included in the univariate Cox regression analysis (GSE44076 had no survival data). Forest plots were drawn *via* GraphPad Prism software (v6.0). Cyclin gene expression in patient tumor samples from the three datasets with survival data (TCGA, GSE39582, and GSE41258) was included in the survival analysis. Survival curves were plotted using the Kaplan-Meier method.

The Cox proportional hazards model (14) was used to construct prediction models based on the expression levels of cyclin family genes and patient overall survival using R software. Accordingly, the risk score of each patient was determined using the following formula:

(1) $$Risk\;score=\sum_{i=1}^{n}Coef_{i}\times Exp_{i}$$

where n is the number of included genes, Coef is the coefficient of each gene, and Exp is the log_2_ - gene expression level.

With the risk scores and overall survival of patients, receiver operating characteristic (ROC) curves were plotted to show the ability to predict five-year survival by the survivalROC package in R. The area under curve (AUC) value on the curve represents predictive accuracy. Patients were divided evenly into high- and low-risk groups according to the risk scores and survival curves were drawn. Prognostic nomograms were generated based on the 24-cyclin prediction model and TNM staging by the rms, foreign, and survival packages in R. The predictive performance of the nomograms was validated by the calibration curves, which compared the nomogram-predicted probabilities of patient survival at three and five years with the observed survival. A plot falls along the 45-degree line on the calibration curve, indicating that the prognostic nomogram possesses good predictive performance (15).

### Analyses of Copy Number Variation (CNV), DNA Methylation, Immune Infiltration, Signaling Pathways, and Drug Sensitivity

The tumor samples of COAD patients were separated into four groups (single deletion, normal, single gain, and amplification) according to the copy number of cyclin genes. Then, the difference in cyclin gene expression among these groups was evaluated by the Kruskal–Wallis test and boxplots were drawn using R. The methylation levels of cyclin genes in the normal and tumor tissues of patients with COAD based on TCGA data were obtained by UALCAN (http://ualcan.path.uab.edu/index.html), a comprehensive and interactive website for analyzing cancer OMICS data.

The Tumor Immune Estimation Resource (https://cistrome.shinyapps.io/timer/) was used to explore the correlations between cyclin genes and six immune cell types (B cells, CD8+ T cells, CD4+ T cells, dendritic cells, macrophages, and neutrophils) in COAD and these were shown using scatterplots with purity-corrected partial Spearman’s rho values and statistical significance.

The pathway activity of cyclin genes was analyzed by GSCA. The potential biological pathways of cyclin genes in colon cancer were investigated by GSEA (16), which is a computational method that determines whether a previously-defined gene set shows significant differences between two biological phenotypes. We dichotomized the tumor samples into high and low expression groups according to the mRNA or protein levels of the critical cyclin gene and analyzed the expression profiles using javaGSEA (http://software.broadinstitute.org/gsea/downloads.jsp; v4.0.3). KEGG gene sets (v7.0), Hallmark gene sets (v7.0), and oncogenic signature gene sets (v7.0) were chosen as references.

The correlation between cyclin gene expression and drug sensitivity was determined using GSCA. Drug sensitivity and gene expression profiling data of cancer cell lines were obtained from the Genomics of Drug Sensitivity in Cancer project (https://www.cancerrxgene.org/). The drugs approved by the Food and Drug Administration (FDA) were included in the analysis.

### Statistical Analysis

The difference in gene expression between tumor samples and normal controls was estimated by the exact test (11) in edgeR and the Empirical Bayes statistical test (17) in limma. The fold-change, *P*-value, and false discovery rate (or adjusted *P*-value) of each gene were determined. Genes with |log_2_[fold-change]| ≥ 0.5 and false discovery rate < 0.05 (or adjusted *P*-value < 0.05) were considered DEGs. SPSS (v23.0) was used to perform univariate Cox regression analysis of patient survival and clinicopathological features. The relationships between gene expression and TNM stage as well as CNV were examined using the Kruskal–Wallis test. Kaplan-Meier survival curves were compared using the log-rank test. The difference in protein levels between normal and tumor samples was determined by the t-test. *P* < 0.05 was considered to indicate significance.

## Results

### Six Cyclin Genes Were Differentially Expressed Between the Normal and Tumor Tissues of Colon Cancer

**Figure 1:**
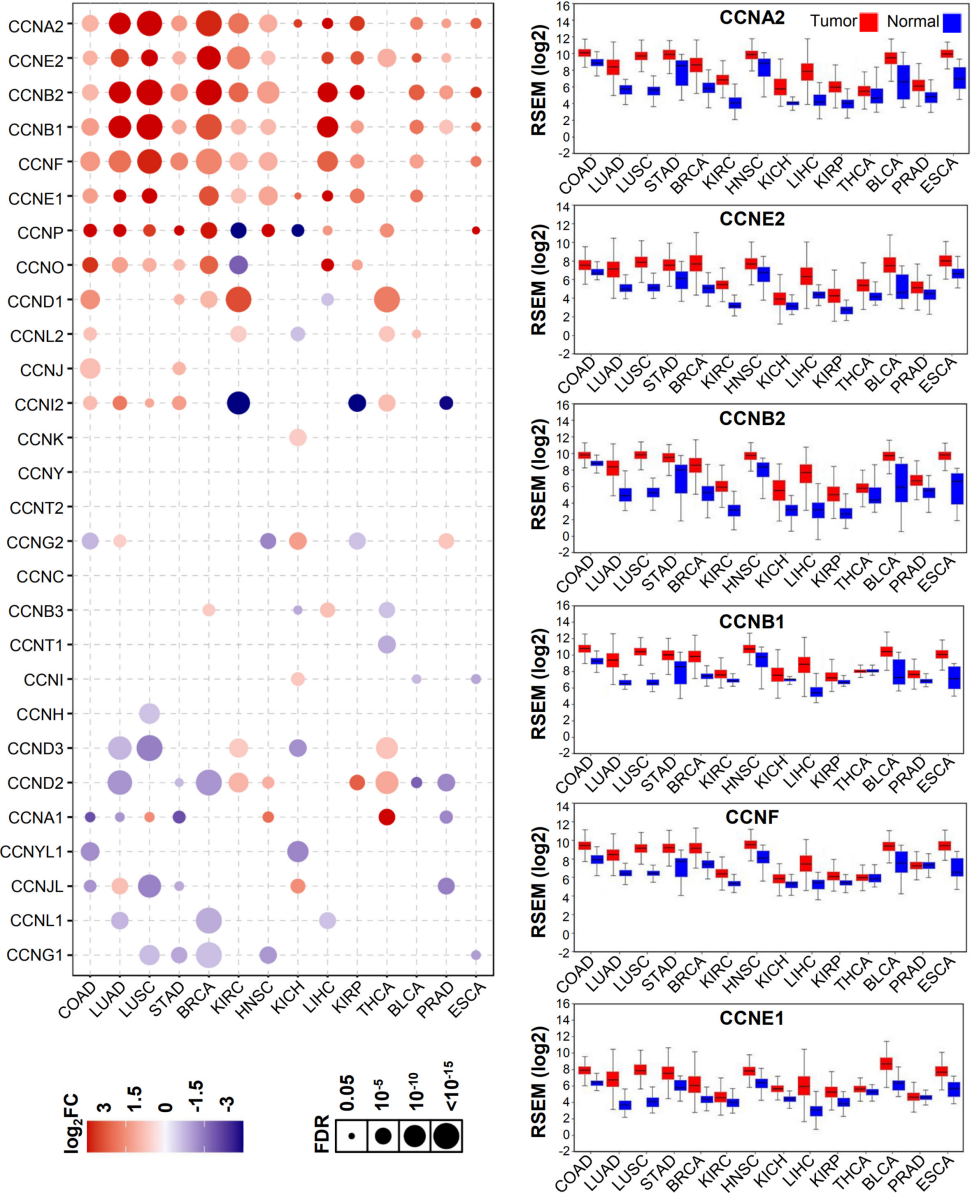
Expression patterns of cyclin family genes between normal and tumor tissues of patients with different cancers. **(A)** The bubble plot shows the expression levels of all cyclin family genes in 14 cancers from GSCA. FC, fold change; FDR, false discovery rate. **(B)** Boxplots of the top six genes in **(A)** from OncoLnc.w.

**Figure 2:**
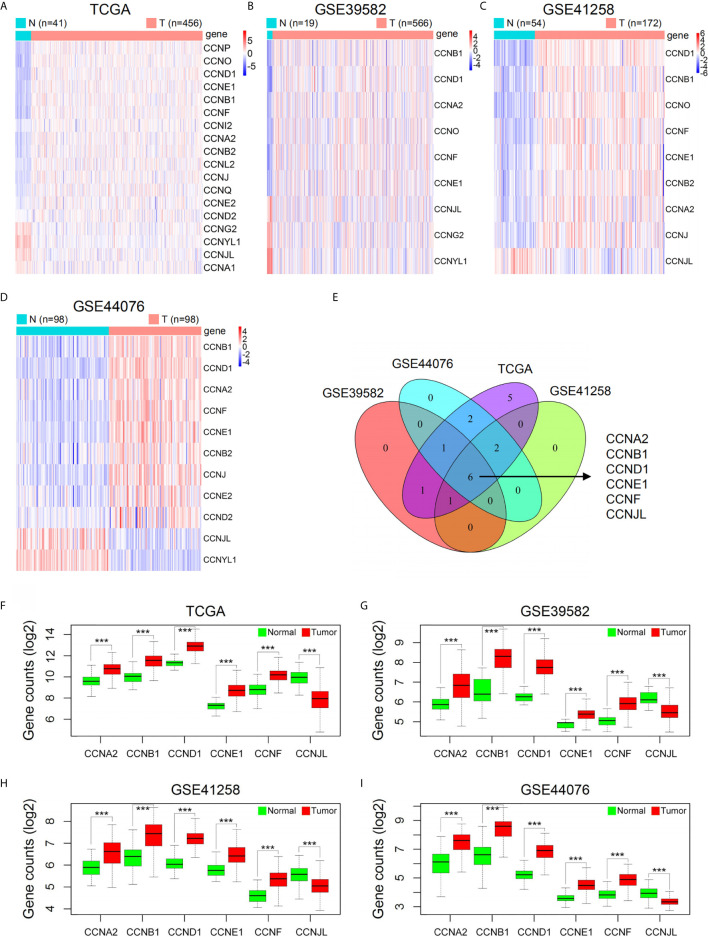
Expression levels of cyclin family genes between normal and tumor samples of patients with colon cancer in the four datasets. **(A–D)** Heatmaps of the DEGs in the cyclin family. The red and blue colors represent high and low expression, respectively. N, normal; T, tumor. **(E)** The Venn diagram indicates six mutual DEGs among the four datasets. **(F–I)** Boxplots of the six DEGs further showed their expression difference between normal and tumor groups. ***P < 0.001.

As shown by GSCA, except *CCNY*, *CCNT2*, and *CCNC*, all cyclin genes were differentially expressed in different cancers, among which the number of genes in COAD was the highest, with 16 genes differentially expressed in total (**Figure 1A**). Among all cyclin genes analyzed, *CCNA2*, *CCNE2*, *CCNB2*, *CCNB1*, *CCNF*, and *CCNE1* were most elevated in the included cancers (**Figure 1B**). Then we performed differential expression analysis in the four datasets of colon cancer. More than half of the cyclin family genes were upregulated in the tumor tissues of patients with colon cancer (**Supplementary Tables 1–4**). Expression patterns of the cyclin DEGs in the four datasets are shown by heatmaps (**Figures 2A–D**). There were six mutual DEGs among the datasets; namely, *CCNA2*, *CCNB1*, *CCND1*, *CCNE1*, *CCNF*, and *CCNJL* (**Figure 2E**), and their expression levels were further presented by boxplots (**Figures 2F–I**). All were upregulated in tumor samples except *CCNJL*, which suggests that high expression of the five genes and low expression of *CCNJL* might be tightly associated with the occurrence of colon cancer.

**Figure 3:**
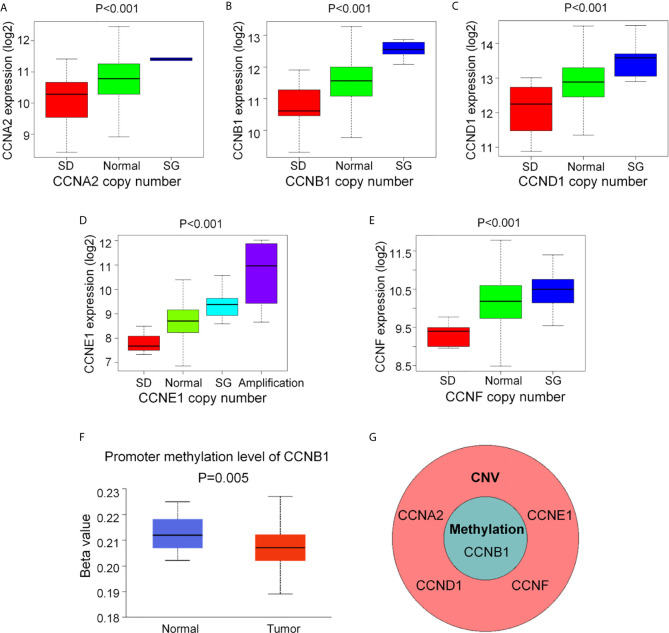
The CNV and DNA methylation of the six cyclin DEGs in patients with COAD from TCGA. **(A–E)** The copy number of *CCNA2*, *CCNB1*, *CCND1*, *CCNE1*, and *CCNF* was positively associated with gene expression. For *CCNJL*, see **Supplementary Figure 1A**. SD, single deletion; SG, single gain. **(F)** The methylation levels of *CCNB1* were noticeably lower in tumor tissues. For the other five genes, see **Supplementary Figures 1B-F**. **(G)** *CCNB1* was the only gene showing significant difference in terms of CNV and methylation. CNV, copy number variation.

After identification of the six cyclin DEGs, we further studied their copy number and methylation to elucidate the regulatory mechanism of gene expression. The tumor samples from TCGA were separated into four groups (single deletion, normal, single gain, and amplification) according to the copy number of cyclin genes. Results revealed that the copy number of *CCNA2*, *CCNB1*, *CCND1*, *CCNE1*, and *CCNF* was significantly positively correlated with their gene expression (*P* < 0.001, **Figures 3A–E**), while *CCNJL* exhibited no significant difference (**Supplementary Figure 1A**). The methylation levels of *CCNB1* were significantly lower in tumor samples (*P* = 0.005, **Figure 3F**), suggesting that its expression might be regulated by DNA methylation in COAD. The other five genes are shown in **Supplementary Figures 1B–F**. *CCNB1* is the only gene that showed significant differences in terms of CNV and methylation (**Figure 3G**).

### Four Cyclin Genes Were Notably Elevated in the Early Clinical Stage and Significantly Associated With Overall Survival of the Patients With Colon Cancer

**Figure 4:**
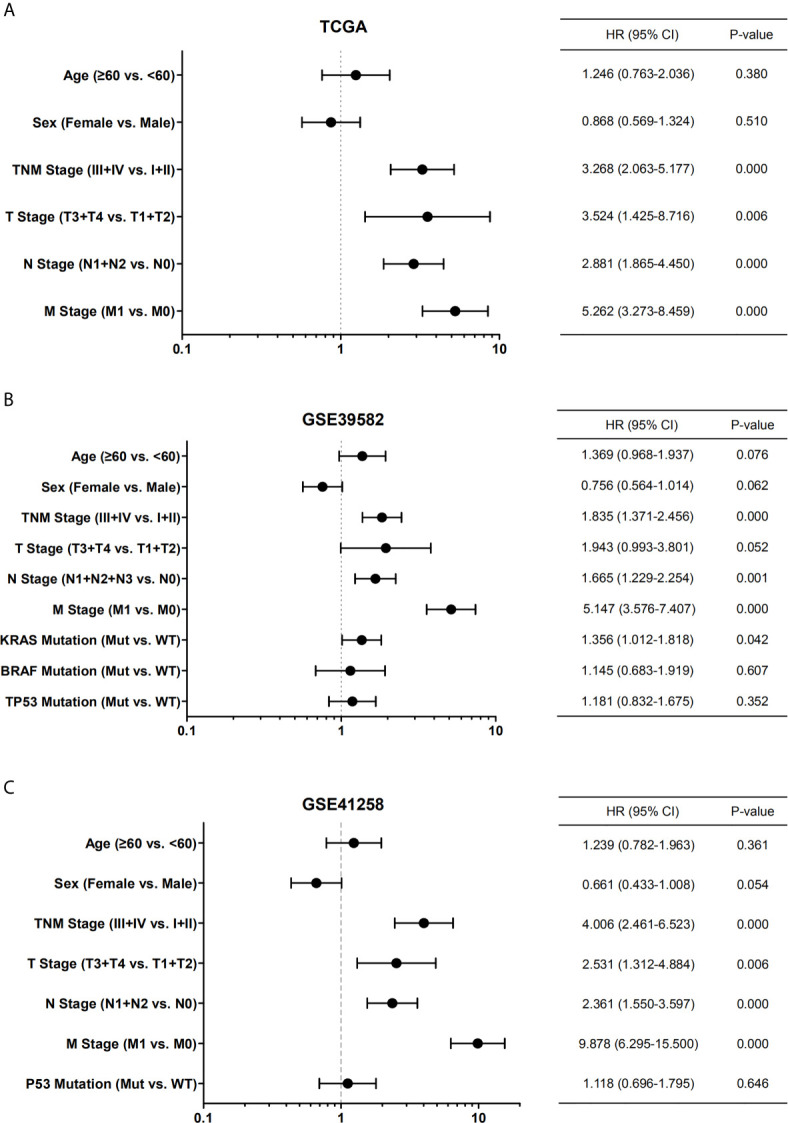
Forest plots showing the univariate Cox regression analysis of overall survival and clinical features of patients with colon cancer from TCGA **(A)**, GSE39582 **(B)**, and GSE41258 **(C)** datasets. Hazard ratio (HR) with confidence interval (CI) is plotted on the x-axis. TNM, tumor-node-metastasis; Mut, mutant; WT, wild type.

**Figure 5:**
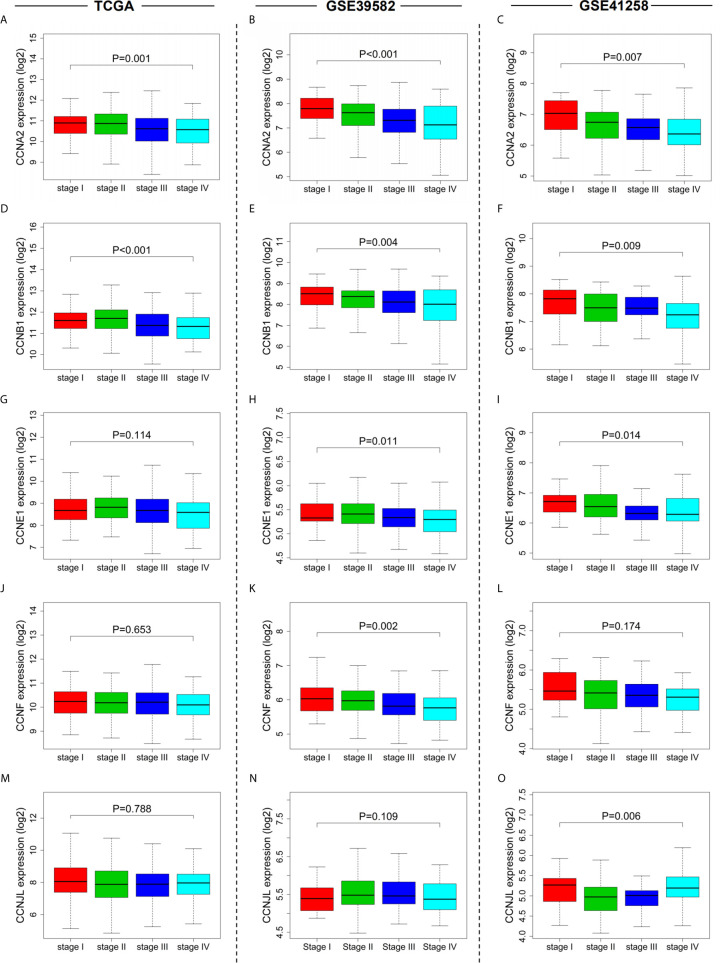
Relationships between cyclin gene expression and patient TNM stages in the three datasets. The expression of *CCNA2* **(A–C)**, *CCNB1* **(D–F)**, *CCNE1* **(G–I)**, *CCNF* **(J–L)**, and *CCNJL* **(M–O)** was significantly associated with patient TNM stages. For *CCND1*, see **Supplementary Figures 2A-C**.

By univariate Cox regression analysis, the hazard ratio with a confidence interval of the clinicopathological features was obtained and presented by forest plots (**Figure 4**). Consequently, patient survival was significantly correlated with TNM, N, and M stages in all three datasets. We then investigated the correlation between the expression of the six cyclin DEGs and the patient TNM stage. As a result, high expression levels of four genes (*CCNA2*, *CCNB1*, *CCNE1*, and *CCNF*) were associated with the early TNM stage (**Figures 5A–L**). *CCNJL* showed inconsistent results among the three datasets (**Figures 5M–O**). No significant difference was observed in *CCND1* in any dataset (**Supplementary Figures 2A–C**). These results indicated that the four cyclin genes were appropriate for the early diagnosis of colon cancer.

**Figure 6:**
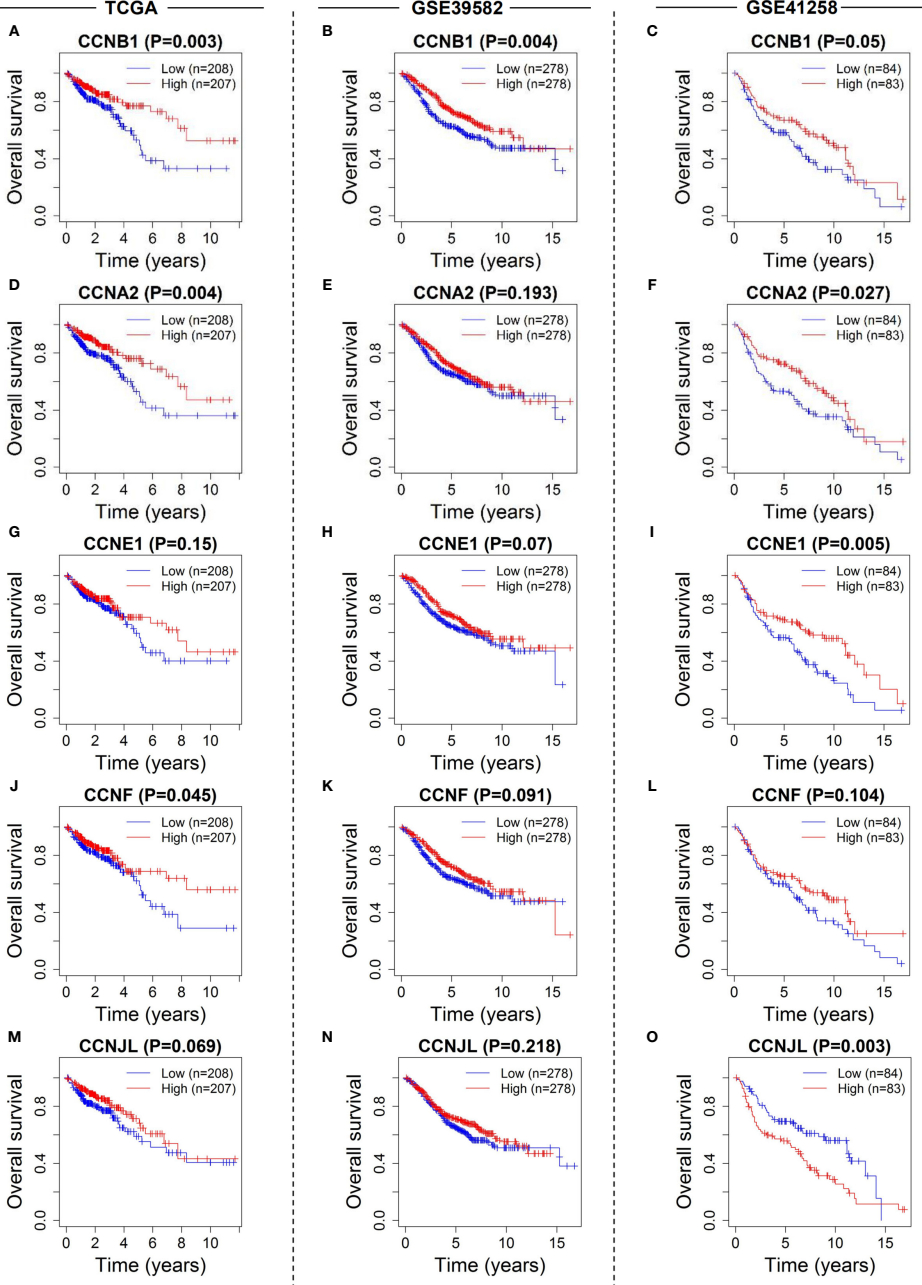
Survival analysis of the six cyclin DEGs in patients with colon cancer in the three datasets. Kaplan-Meier survival curves showed that expression of *CCNA2* **(A–C)**, *CCNB1* **(D–F)**, *CCNE1* **(G–I)**, *CCNF* **(J–L)**, and *CCNJL* **(M–O)** was significantly related to patient survival. For *CCND1*, see **Supplementary Figures 2D-F**.

Survival curves showed that five cyclin DEGs (*CCNA2*, *CCNB1*, *CCNE1*, *CCNF*, and *CCNJL*) had significant correlations with the overall survival of patients in at least one dataset (**Figures 6A–L**). In particular, *CCNB1* was significantly correlated with patient survival in all three datasets (*P* = 0.003 in TCGA, **Figure 6A**; *P* = 0.004 in GSE39582, **Figure 6B**; *P* = 0.050 in GSE41258, **Figure 6C**), indicating that *CCNB1* might be the most important cyclin gene for the prognosis of colon cancer. *CCNJL* did not show coincident results among the three datasets (low expression correlated with poor survival in TCGA and GSE39582 but with favorable prognosis in GSE41258, **Figures 6M–O**). No significant difference was observed in *CCND1* in any dataset (**Supplementary Figures 2D–F**). Overall, patients with lower expression of the four genes (*CCNA2*, *CCNB1*, *CCNE1*, and *CCNF*) had worse survival outcomes, which implies that these members of the cyclin family might be ideal prognostic biomarkers for colon cancer.

### Prediction Models of Cyclin Family Genes and TNM Staging

**Figure 7:**
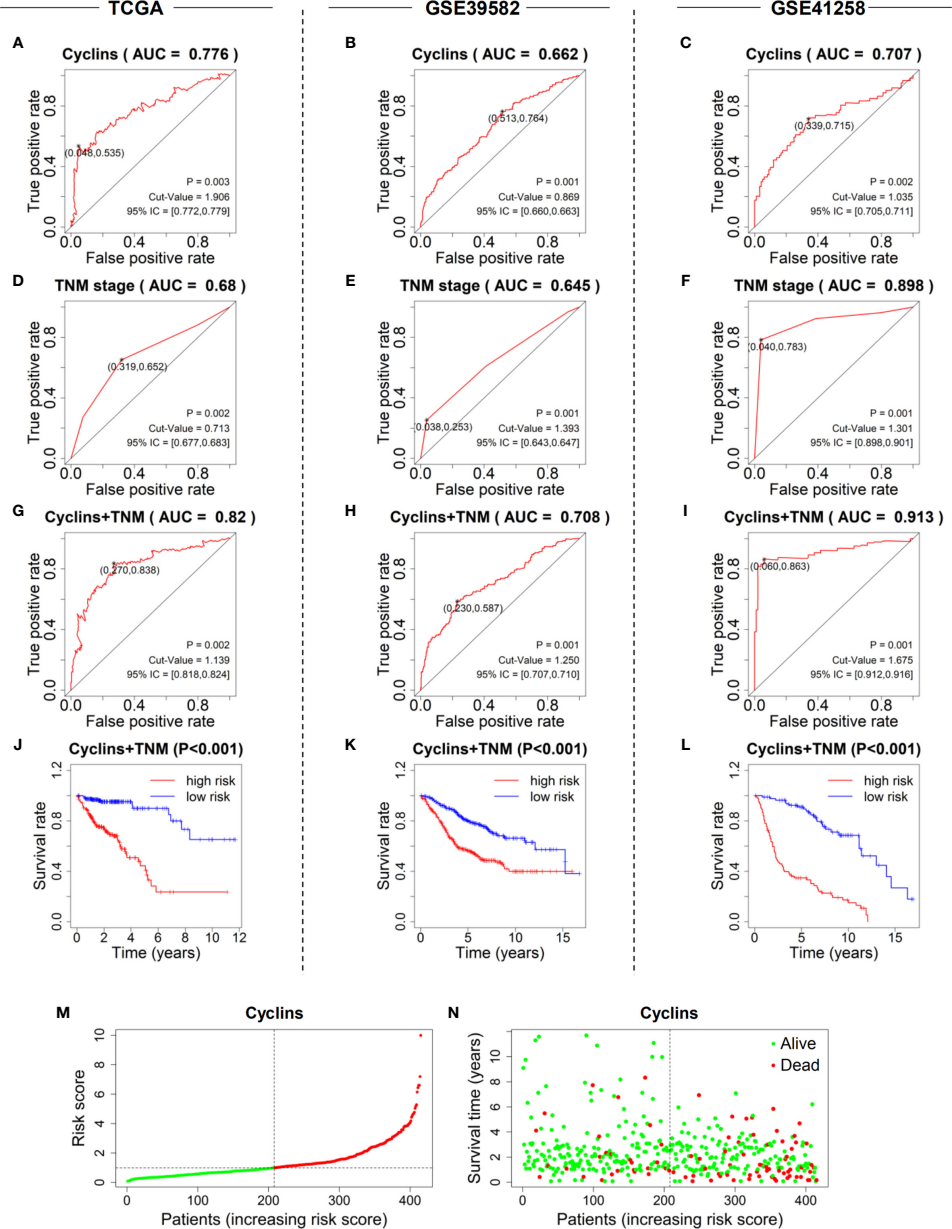
Prediction models to predict the prognosis of patients with colon cancer in the three datasets. **(A–C)** Five-year ROC curves of the prediction models based on the expression of 24 cyclin genes. **(D–F)** Five-year ROC curves of the prediction models based on patient TNM stage. **(G–I)** Five-year ROC curves of the prediction models integrating 24 cyclin genes and TNM stage. **(J–L)** Survival curves showing the survival difference between high- and low-risk patients. The risk scores were calculated by the prediction models integrating 24 cyclin genes and TNM stage. **(M)** Risk score distribution of the patients in ascending order and divided into low- (green) and high-risk (red) in TCGA dataset. **(N)** Survival time and status of the patients in order of increasing risk scores in TCGA dataset. Red and green dots represent dead and alive, respectively.

To assess the predictive values of cyclin genes in colon cancer, we established prediction models based on the expression levels of 24 cyclin genes in the three datasets. An AUC value above 0.6 indicates the model has predictive ability (18). Eventually, the AUC of the 24-cyclin prediction model reached 0.776 (**Figure 7A**), 0.662 (**Figure 7B**), and 0.707 (**Figure 7C**) in TCGA, GSE39582, and GSE41258 datasets, respectively, all exceeding 0.6. In particular, the established model of cyclins possessed higher predictive accuracy than the TNM staging-based model in TCGA and GSE39582 datasets (**Figures 7D, E**), but not in GSE41258 dataset (**Figure 7F**). We further combined cyclin genes with TNM staging, which was more accurate than TNM staging only (**Figures 7G–I**). Survival curves indicated that the survival rates of high-risk patients were considerably lower than those of low-risk patients (**Figures 7J–L**). According to the risk scores generated by the 24-cyclin model in TCGA dataset, the risk score distribution and survival time of patients were presented in order of increasing risk score (**Figures 7M, N**). Patients with a high risk score had higher mortality rates than those with a low risk score (**Figure 7N**). Taken together, cyclin genes might have stronger predictive ability than TNM staging.

**Figure 8:**
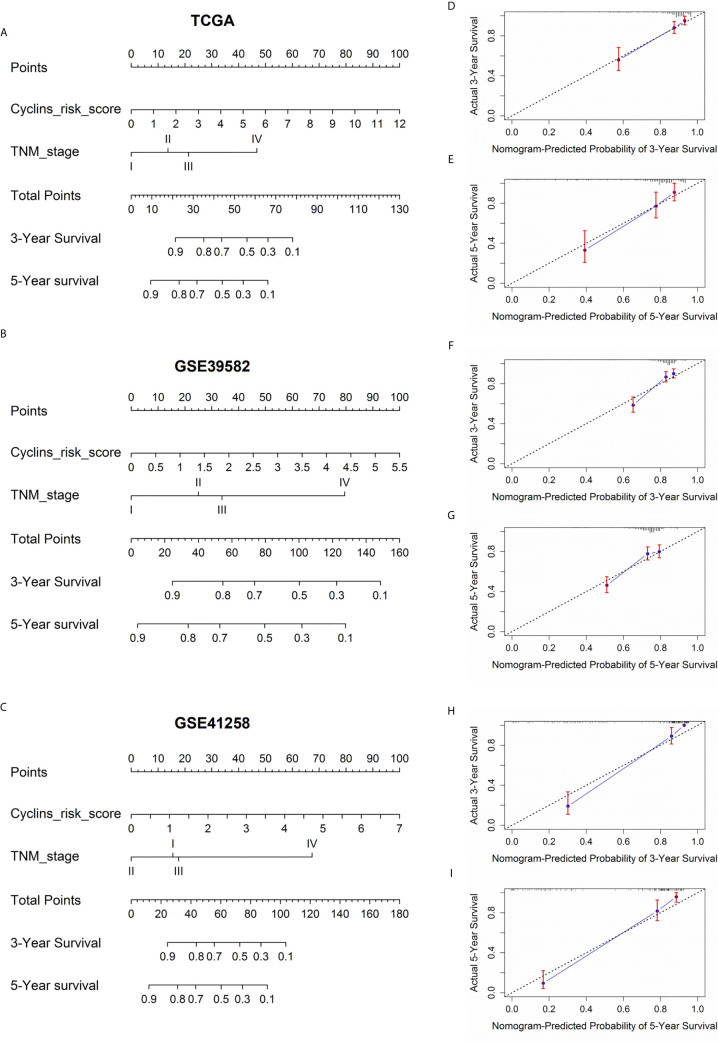
Nomograms to predict the probability of three- and five-year survival in patients with colon cancer. **(A–C)** Prognostic nomograms according to the risk score of the prediction models integrating 24 cyclin genes and TNM stage. The value on the lines of each variable corresponds to a point on the “Points” line. The sum of these points on the “Total Points” line corresponds to the probability of three- and five-year survival. **(D–I)** Calibration curves indicated the predictive accuracy of the nomograms. The black dotted line is a reference line. Red bars and blue lines represent error bars and calibration lines, respectively.

To develop a practical method to predict patient survival, nomograms were constructed by integrating patient risk scores calculated by the 24-cyclin prediction model with TNM staging (**Figures 8A–C**). Each variable had a score, and the probability of three- and five-year survival could be predicted by the sum of these scores. Calibration curves demonstrated that the nomogram-predicted probability of three- and five-year survival was highly consistent with the actual survival rates (**Figures 8D–I**). Collectively, a combination of cyclin genes and TNM staging may be useful for the accurate survival prediction of patients with colon cancer.

### Immune Infiltration Analysis of the Four Significant Cyclin Genes

**Figure 9:**
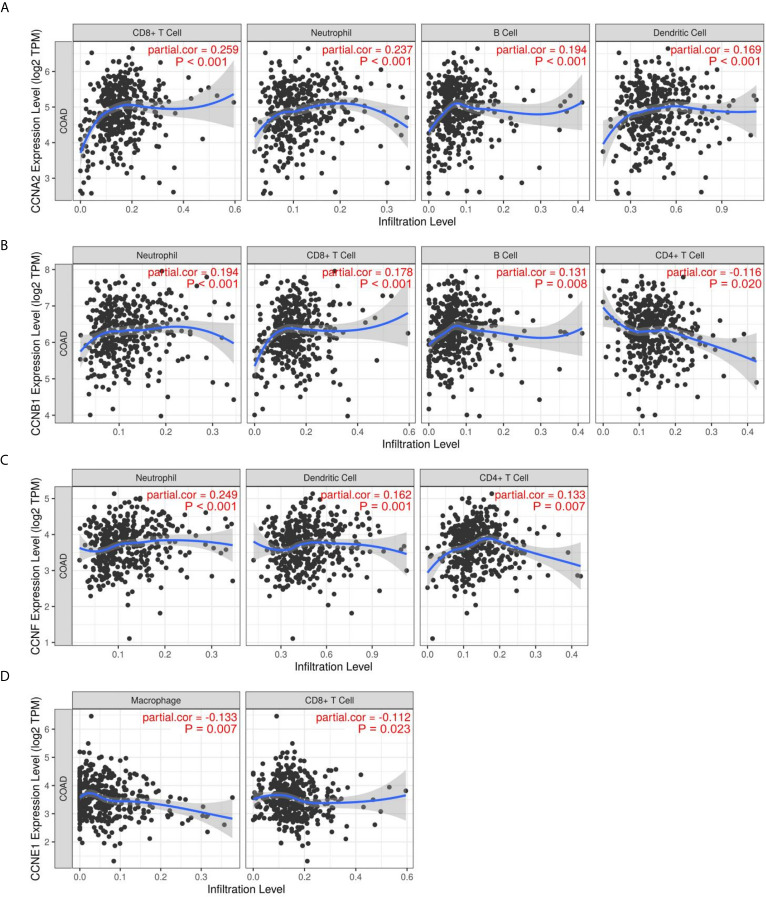
Immune infiltration of the four significant cyclin genes in colon cancer. **(A–D)** *CCNA2*, *CCNB1*, *CCNE1*, and *CCNF* were significantly correlated with immune cells, including CD8+ T cells, CD4+ T cells, neutrophils, B cells, dendritic cells, and macrophages.

The correlations between cyclin genes and immune cells in COAD were explored using the Tumor Immune Estimation Resource. *CCNA2* was significantly associated with CD8+ T cells, neutrophils, B cells, and dendritic cells (**Figure 9A**). *CCNB1* was related to neutrophils, CD8+ T cells, B cells, and CD4+ T cells (**Figure 9B**). *CCNF* was positively correlated with neutrophils, dendritic cells, and CD4+ T cells (**Figure 9C**), while *CCNE1* was negatively correlated with macrophages and CD8+ T cells (**Figure 9D**). These results suggest that *CCNA2* and *CCNB1* might positively regulate tumor-killing immune cells, such as CD8+ T cells, thereby prolonging the survival time of patients with colon cancer.

### Pathway Analysis and Co-Expression Network of the Four Significant Cyclin Genes

**Figure 10:**
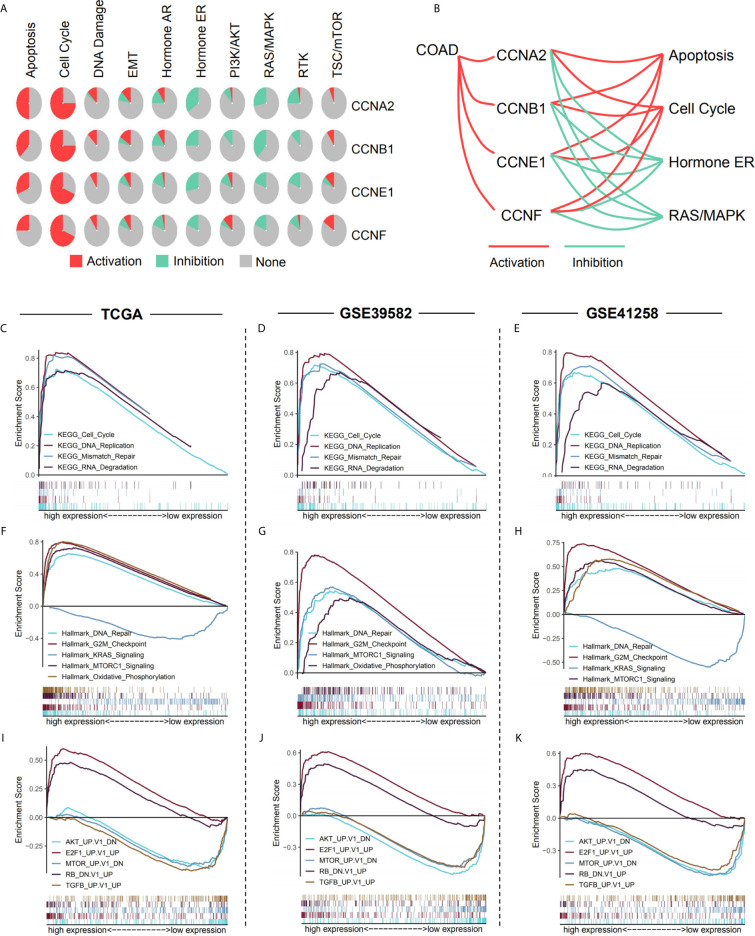
Pathway analysis of the four significant cyclin genes. **(A)** Pathway activity of the four genes from GSCA. **(B)** *CCNA2*, *CCNB1*, *CCNE1*, and *CCNF* might strongly activate the cell cycle and apoptosis as well as inhibit hormone ER and RAS/MAPK pathways. **(C–K)** Enriched gene sets between high and low expression sample groups based on the *CCNB1* gene levels in the three datasets using GSEA. Reference gene sets were KEGG **(C–E)**, Hallmark **(F–H)**, and oncogenic signatures gene sets **(I–K)**.

Analysis of pathway activity by GSCA revealed that *CCNA2*, *CCNB1*, *CCNE1*, and *CCNF* might strongly activate the cell cycle and apoptosis as well as inhibit hormone ER and RAS/MAPK pathways (**Figures 10A, B**). Since *CCNB1* was the only gene that showed a significant correlation with overall survival in the three datasets and its expression was highly correlated with the other three genes, *CCNA2*, *CCNE1*, and *CCNF* (**Supplementary Figure 3**), we performed GSEA based on the expression levels of *CCNB1* to identify the related pathways and underlying mechanisms. Results showed that most of the gene sets were upregulated in the high *CCNB1* expression group, including the cell cycle (**Figures 10C–E**), DNA replication (**Figures 10C–E**), DNA repair (**Figures 10F–H**), and G2/M checkpoint (**Figures 10F–H**), which are consistent with the known functions of cyclins. Gene sets of mTORC1 signaling, KRAS signaling, and oxidative phosphorylation were also significantly enriched (**Figures 10F–H**), which may unravel the process of cyclin genes (especially *CCNB1*) in colon cancer. Additionally, genes related to Akt, mTOR, and TGFB were enriched in the low *CCNB1* expression group (**Figures 10I–K**).

**Figure 11:**
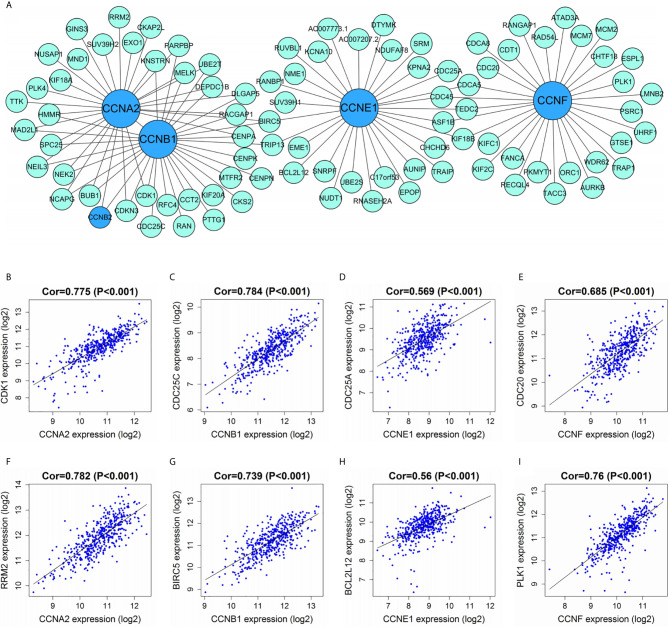
Correlations between cyclin genes and other DEGs in TCGA dataset. **(A)** Co-expression network of the four significant cyclin genes and their co-expressed genes. The top 30 genes most correlated with each of them are presented. **(B–E)** The four cyclin genes were tightly linked to cell cycle-relevant genes (*CDK1*, *CDC25C*, *CDC25A*, and *CDC20*). **(F–I)** The four cyclin genes had close connections with the mTOR- (*RRM2*) and cancer-related genes (*BIRC5*, *BCL2L12*, and *PLK1*). Cor, correlation coefficient.

WGCNA was carried out to identify the genes co-expressed with the four important cyclin genes (*CCNA2*, *CCNB1*, *CCNE1*, and *CCNF*) in TCGA dataset (**Figure 11A**). *CCNA2* and *CCNB1* were tightly linked to each other and co-expressed with several genes in common, including *CCNB2*, *CDK1*, *BUB1*, *NEK2*, and *CENPA*. The four cyclin genes were closely connected with cell cycle-relevant genes (*CDK1*, *CDC25C*, *CDC25A*, and *CDC20*; **Figures 11B–E**), as well as mTOR-related gene (RRM2) and cancer-related genes (*BIRC5*, *BCL2L12*, and *PLK1*; **Figures 11F–I**).

### Protein Expression of the Four Significant Cyclin Genes

**Figure 12:**
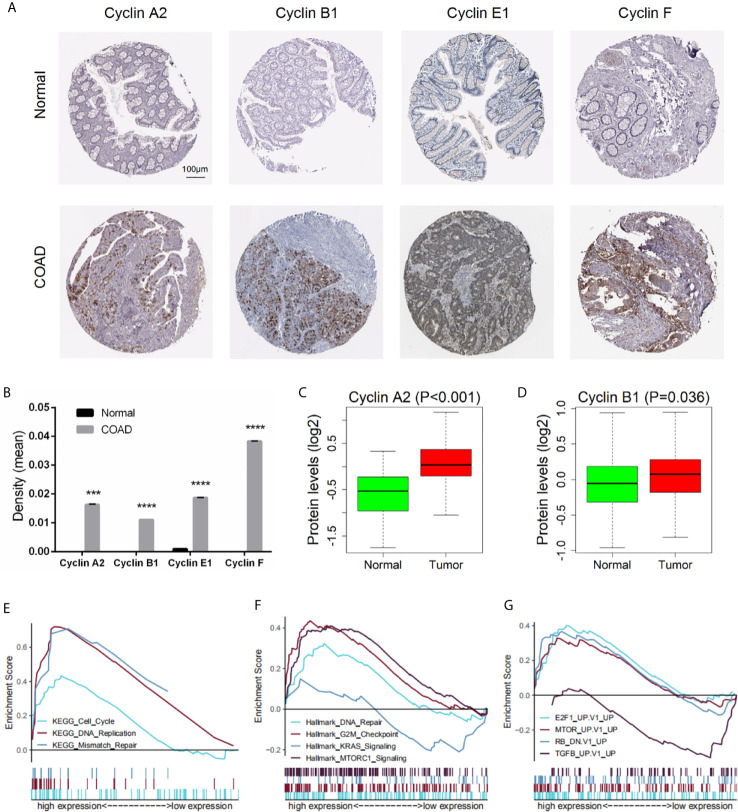
Protein expression of the four significant cyclin genes in COAD. **(A)** Immunohistochemical images of the four cyclins from the HPA. **(B)** Protein expression density of Cyclin A2, Cyclin B1, Cyclin E1, and Cyclin F were higher in tumor than in normal tissues from the HPA. ***P < 0.001, ****P < 0.0001. **(C, D)** The protein levels of Cyclin A2 and Cyclin B1 were elevated in tumor compared to normal samples from the CPTAC database (Cyclin E1 and Cyclin F were not available). **(E–G)** GSEA results based on the protein levels of Cyclin B1 from CPTAC.

To investigate the protein expression of cyclins, we collected data from the HPA and CPTAC databases. The immunohistochemical images of the four cyclins in normal colon tissues and COAD were from the HPA (**Figure 12A**). The measurements of expression density indicated that Cyclin A2, Cyclin B1, Cyclin E1, and Cyclin F had higher expression in COAD than in normal tissues (**Figure 12B**). Patient information is provided in **Supplementary Table 5**. Analysis of the data from CPTAC showed that the protein levels of Cyclin A2 and Cyclin B1 were elevated in tumors compared to normal samples (**Figures 12C, D**). Cyclin E1 and Cyclin F were not available. The protein expression profiles obtained from CPTAC were also submitted to GSEA and the enriched gene sets were similar to those based on the gene expression profiles (**Figures 12E–G**).

### Drug Sensitivity Analysis of the Four Significant Cyclin Genes

**Figure 13:**
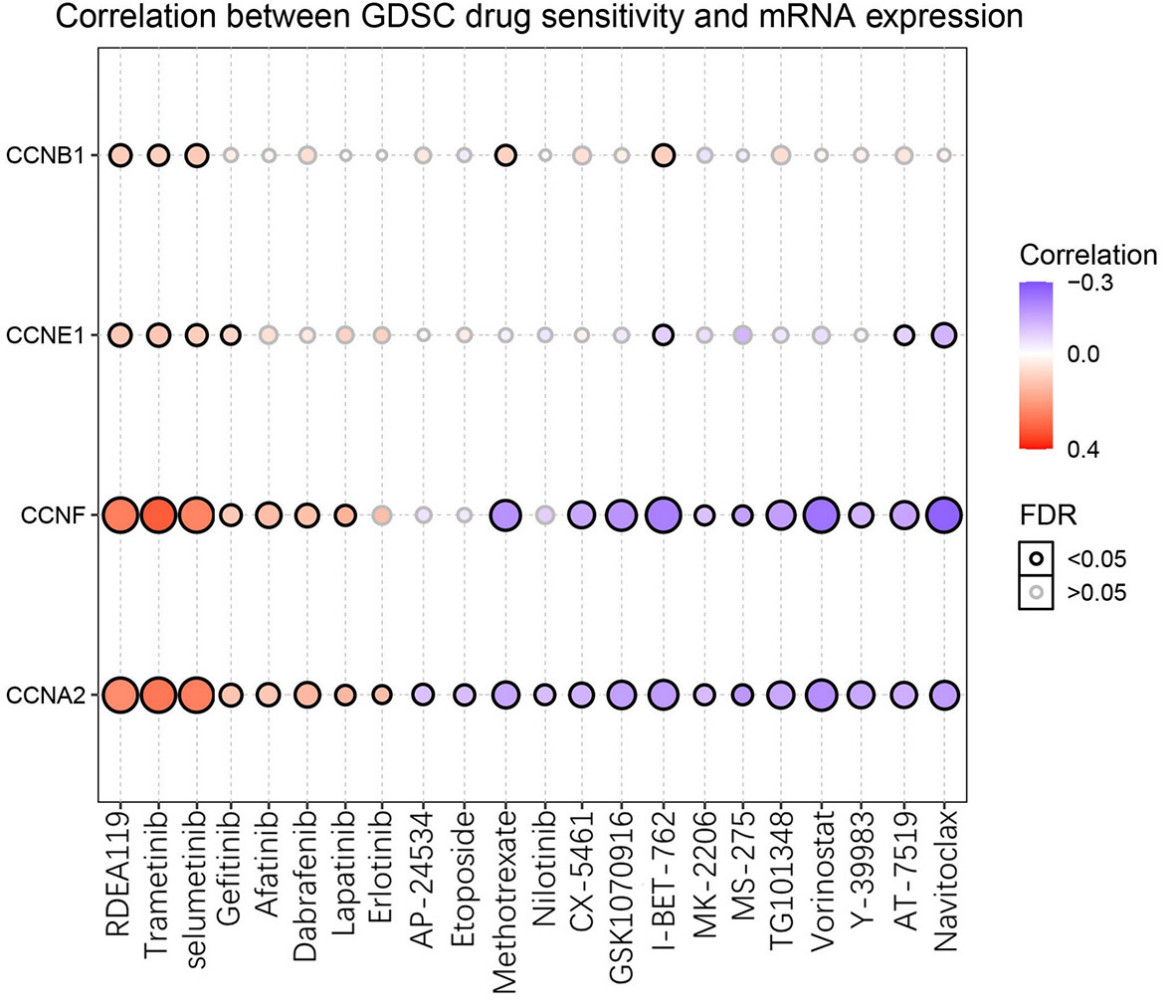
Drug sensitivity of the four significant cyclin genes from GSCA. The bubble plot exhibits the correlations between the gene expression and FDA-approved drugs. The positive Spearman correlation coefficients indicate that high gene expression is resistant to the drug, and *vice versa*. GDSC, Genomics of Drug Sensitivity in Cancer.

After GSCA analysis, the drug sensitivity of *CCNA2*, *CCNB1*, *CCNE1*, and *CCNF* was shown by a bubble plot (**Figure 13**). High expression of *CCNB1* was associated with drug resistance. Besides, both high and low expression of *CCNA2*, *CCNE1*, and *CCNF* were correlated with drug resistance depending on the type of drug used. High expression of the four genes might be resistant to RDEA119, trametinib, and selumetinib, which are all MEK inhibitors (19–21). In addition, high expression of *CCNA2* and *CCNF* was thought to be resistant to gefitinib, afatinib, and lapatinib, which are EGFR inhibitors (22–24). Conversely, high expression of *CCNA2* and *CCNF* might be sensitive to certain drugs, such as methotrexate (DHFR inhibitor), vorinostat (HDAC inhibitor), and navitoclax (Bcl-2 inhibitor) (25–27).

## Discussion

Dysregulation of the cell cycle control may lead to tumor progression (28). Cyclins, proteins that regulate the cell cycle, are associated with a wide range of cancer types (7). By analyzing the expression profiles of cyclin genes in different types of cancers through GSCA, we found that cyclin genes were most differentially expressed in COAD. However, the roles of most cyclin family members and their regulatory mechanisms in colon cancer remain unclear. In recent years, next-generation sequencing technology has seen great advances and has been widely applied in the field of oncology (29). Therefore, we adopted a series of bioinformatics methods to analyze the RNA sequence data of colon cancer tissues to provide a systematic and comprehensive analysis of cyclin family genes in colon cancer.

In this study, the differential expression analysis showed that among all cyclin family members, there were six significant DEGs (*CCNA2*, *CCNB1*, *CCND1*, *CCNE1*, *CCNF*, and *CCNJL*), five of which were overexpressed in tumor samples compared to normal controls, while *CCNJL* was downregulated. This suggests that these genes might be tightly associated with the occurrence of colon cancer and are likely to be diagnostic markers for patients with colon cancer. Consistently, *CCNA2*, *CCNB1*, *CCND1*, and *CCNF* have been observed to be upregulated in colorectal cancer (30–33), but there is no clear evidence that *CCNE1* and *CCNJL* are also differentially expressed between colon tumor and normal tissues. By comparing the expression levels of these DEGs among patients with different TNM stages, we found that *CCNA2*, *CCNB1*, *CCNE1*, and *CCNF* had particularly higher expression in patients with stage I colon cancer than in those with the advanced stage, implying that they are suitable for the diagnosis of early stage colon cancer.

Apart from gene expression, we studied the copy number and DNA methylation of the six cyclin DEGs. Analysis of the correlation between CNV and gene expression showed that the copy number of *CCNA2*, *CCNB1*, *CCND1*, *CCNE1*, and *CCNF* was positively related to gene expression. Toncheva et al. concluded that *CCND1* gene amplification was a possible cause of protein overexpression in colorectal cancer (34), which is in accordance with our findings. Among the six genes, the methylation levels of *CCNB1* were lower in tumor than in normal tissues and were negatively correlated with its gene expression, as it was overexpressed in tumor tissues. These results suggest that the gene expression of *CCNB1* might be regulated by copy number and DNA methylation in COAD. These specific regulatory mechanisms further provide reliable evidence for the differential gene expression of cyclins.

Next, we explored the relationship between cyclin gene expression and patient survival. Kaplan-Meier survival curves indicated that the six DEGs excluding *CCND1* were significantly associated with patient overall survival in at least one dataset. Among them, the curve of *CCNJL* in GSE41258 contrasted with that in the other two datasets, possibly because *CCNJL* expression was relatively lower in tumor tissues, which led to inaccuracies. Therefore, the other four genes (*CCNA2*, *CCNB1*, *CCNE1*, and *CCNF*) could be appropriate indicators of prognosis, which implies that patient survival might be estimated by detecting the gene expression levels. As illustrated by the survival curves, patients with lower levels of these genes showed poorer survival rates. A previous study also showed that high expression of *CCNB1* and *CCNF* was significantly correlated with favorable prognosis in colorectal cancer, which is in agreement with our findings (33). However, it seems that the significance of *CCNA2* and *CCNE1* for the prognosis of colon cancer hasn’t been elucidated yet. Additionally, the immune infiltration analysis showed that the expression of *CCNA2* and *CCNB1* was positively correlated with CD8+ T cells. In the tumor microenvironment, CD8+ T cells can be activated and help kill cancer cells (35); thus, prolonging the patient survival time. Therefore, it is reasonable that the high expression of cyclin genes is associated with a good prognosis. To assess the predictive values of cyclin genes in colon cancer, we constructed prediction models based on the expression of 24 cyclin genes using the Cox proportional hazards model. Importantly, the ROC curves of these models each had an AUC value over 0.6, indicating that they all possessed predictive ability. In TCGA and GSE39582 datasets, the AUCs of the 24-cyclin-based models were higher than those of the TNM-based models, indicating that cyclin genes might have better predictive accuracy than TNM staging. Moreover, a combination of the 24 cyclin genes and TNM staging exhibited much higher predictive accuracy compared to TNM staging only. Therefore, it may be useful to predict the survival rates of patients with colon cancer by their TNM stage together with cyclin gene expression. With the prognostic nomograms, it is possible to score each patient and predict the three- and five-year survival rates according to the cyclin-based prediction model and the patient’s TNM stage. The calibration curves showed that the nomogram-predicted probability of three- and five-year survival was highly consistent with the actual survival rates, which signifies that our prediction model is reliable and feasible.

GSEA was conducted based on the expression levels of *CCNB1*, as it was the only gene significantly correlated with overall survival in all three datasets and was closely related to the other three genes (*CCNA2*, *CCNE1*, and *CCNF*). Results revealed that gene sets of the cell cycle, DNA replication, mismatch repair, DNA repair, and G2/M checkpoint were enriched in the *CCNB1* high expression group, coinciding with the known functions of cyclins (5, 7, 36, 37). Interestingly, DNA mismatch repair deficiency, which is characterized by the loss of mismatch repair pathway function, frequently occurs in colorectal cancer (38). Thus, we assumed that cyclin genes might regulate the activation of mismatch repair in colon cancer. The enriched gene sets also included mTORC1 signaling, mTOR, and Akt. The mTOR signaling pathway is involved in the onset and progression of cancer, and the mTOR complex (mTORC1 and mTORC2) exerts its actions by regulating some important kinases, such as Akt (39). In addition, the KRAS signaling pathway and TGFB-related genes were upregulated in the low expression group. KRAS is usually mutated in colorectal cancers (40), and the KRAS and TGF pathways are responsible for tumor progression (41). In addition, genes relevant to E2F1 and retinoblastoma (RB) are enriched in the high expression group. E2F1, a transcription factor, is a downstream target of G1 CDK activity (42). Moreover, E2F is a functional target for the action of RB, which is considered a tumor suppressor (43). These results indicate that *CCNB1* is involved in diverse biological processes, many of which have close connections with tumor development and progression.

Given the potential of the four important cyclin genes (*CCNA2*, *CCNB1*, *CCNE1*, and *CCNF*), we further investigated the genes co-expressed with them in colon cancer *via* WGCNA. As a result, cyclin genes were closely linked to several genes that participate in the cell cycle, such as *CDK1*, *CDC25C*, *CDC25A*, and *CDC20* (44). In particular, the Cyclin B1-CDK1 complex is a major component of the G2/M checkpoint (45). *CCNA2*, *CCNB1*, and *CCNE1* were co-expressed with *BIRC5*, a well-known cancer therapeutic target (46) which directly regulates both apoptosis and mitosis in cancer cells during tumorigenesis and tumor metastasis (47). In addition, there was a strong connection between *CCNA2* and *RRM2*. mTOR enhances the transcription of *RRM1* and *RRM2* (48). *CCNE1* and *CCNF* were respectively associated with *BCL2L12* and *PLK1*. *BCL2L12* is a potential new biomarker for colon cancer (49) and its transcript variant BCL2L12A might be a cell cycle regulator that interferes with G2-M transition (50). PLK is required for multiple mitotic processes in mammalian cells (51). More importantly, high *PLK1* expression significantly improved the survival of patients with colon cancer expressing a truncated APC (52). These results suggest that *CCNA2*, *CCNB1*, *CCNE1*, and *CCNF* are associated with many cancer-relevant genes, providing insight into the functions of the cyclin family.

The drug sensitivity analysis illustrated that high expression of the four cyclin genes (*CCNA2*, *CCNB1*, *CCNE1*, and *CCNF*) was related to the drug resistance of several MEK and EGFR inhibitors, which means that patients with high expression of these cyclin genes might be resistant to MEK or EGFR inhibitors. Thus, it is better to not use MEK or EGFR inhibitors for the clinical treatment of patients with high expression of these cyclin genes. In contrast, high expression of *CCNA2* and *CCNF* might be sensitive to certain drugs, such as methotrexate (DHFR inhibitor), vorinostat (HDAC inhibitor), and navitoclax (Bcl-2 inhibitor). Using these drugs to treat the patients with high expression of CCNA2 or CCNF may be more effective.

However, there were some limitations to our study. For example, owing to the limitation of manuscript length, we only undertook research on COAD, not lung adenocarcinoma, lung squamous cell carcinoma, or breast carcinoma, although these are also important. Besides, the transcriptome data were relatively comprehensive, but the proteome data in CPTAC were incomplete, resulting in the insufficient study of cyclins at the protein level. Moreover, considering that our research objective was to provide a comprehensive analysis of cyclin family members, we did not carry out cell experiments to knockdown a single gene *via* siRNA to confirm the results.

## Conclusion

In this study, we identified six cyclin genes (*CCNA2*, *CCNB1*, *CCND1*, *CCNE1*, *CCNF*, and *CCNJL*) that are potential diagnostic biomarkers of colon cancer. Four of them (*CCNA2*, *CCNB1*, *CCNE1*, and *CCNF*) are promising for early diagnosis and prognosis. Additionally, a combination of cyclin genes and TNM stage possesses higher predictive accuracy in patient prognosis than the TNM stage only. Cyclin gene expression in colon cancer might be regulated by DNA methylation and copy number. The expression of *CCNA2* and *CCNB1* was also positively correlated with tumor-killing immune cells, such as CD8+ T cells. During the progression of colon cancer, cyclin genes might be tightly associated with the cell cycle, hormone ER, the RAS/MAPK pathway, mismatch repair, mTORC1 signaling, KRAS signaling, etc., and they are closely correlated with *BIRC5*, *PLK1*, and *BCL2L12*. Furthermore, the high expression of cyclin genes was related to the drug resistance of several MEK and EGFR inhibitors.

## Data Availability Statement

Publicly available datasets were analyzed in this study. This data can be found here: TCGA (https://portal.gdc.cancer.gov/repository), GSE39582 (https://www.ncbi.nlm.nih.gov/geo/query/acc.cgi?acc=GSE39582), GSE41258 (https://www.ncbi.nlm.nih.gov/geo/query/acc.cgi?acc=GSE41258), and GSE44076 (https://www.ncbi.nlm.nih.gov/geo/query/acc.cgi?acc=GSE44076).

## Author Contributions

JL, LZ, and SW conceived and designed the study. JL and LZ analyzed the data and prepared the manuscript. LY, DJ, and KL participated in data collection and analysis. XW gave major advice on the revision of manuscript. All authors contributed to the article and approved the submitted version.

## Funding

This research was funded by National Natural Science Foundation of China (82073937), Natural Science Foundation of Guangdong Province (2018A030313122), Shenzhen Science and Technology Project (JCYJ20180305163658916, JCYJ20180228175059744), Shenzhen Key Medical Discipline Construction Fund (SZXK059), and SZU Medical Young Scientists Program (No.71201-000001).

## Conflict of Interest

The authors declare that the research was conducted in the absence of any commercial or financial relationships that could be construed as a potential conflict of interest.
