# Supplementary material for: Comprehensive Analysis of Cyclin Family Gene Expression in Colon Cancer
Source: Front Oncol. 2021 Apr 29;11:674394. doi: 10.3389/fonc.2021.674394 (PMC8117346; doi:10.3389/fonc.2021.674394)
Supplement: Supplementary file 1 [file DataSheet_1.docx]

Supplementary Material

# Supplementary Figures and Tables

## Supplementary Figures


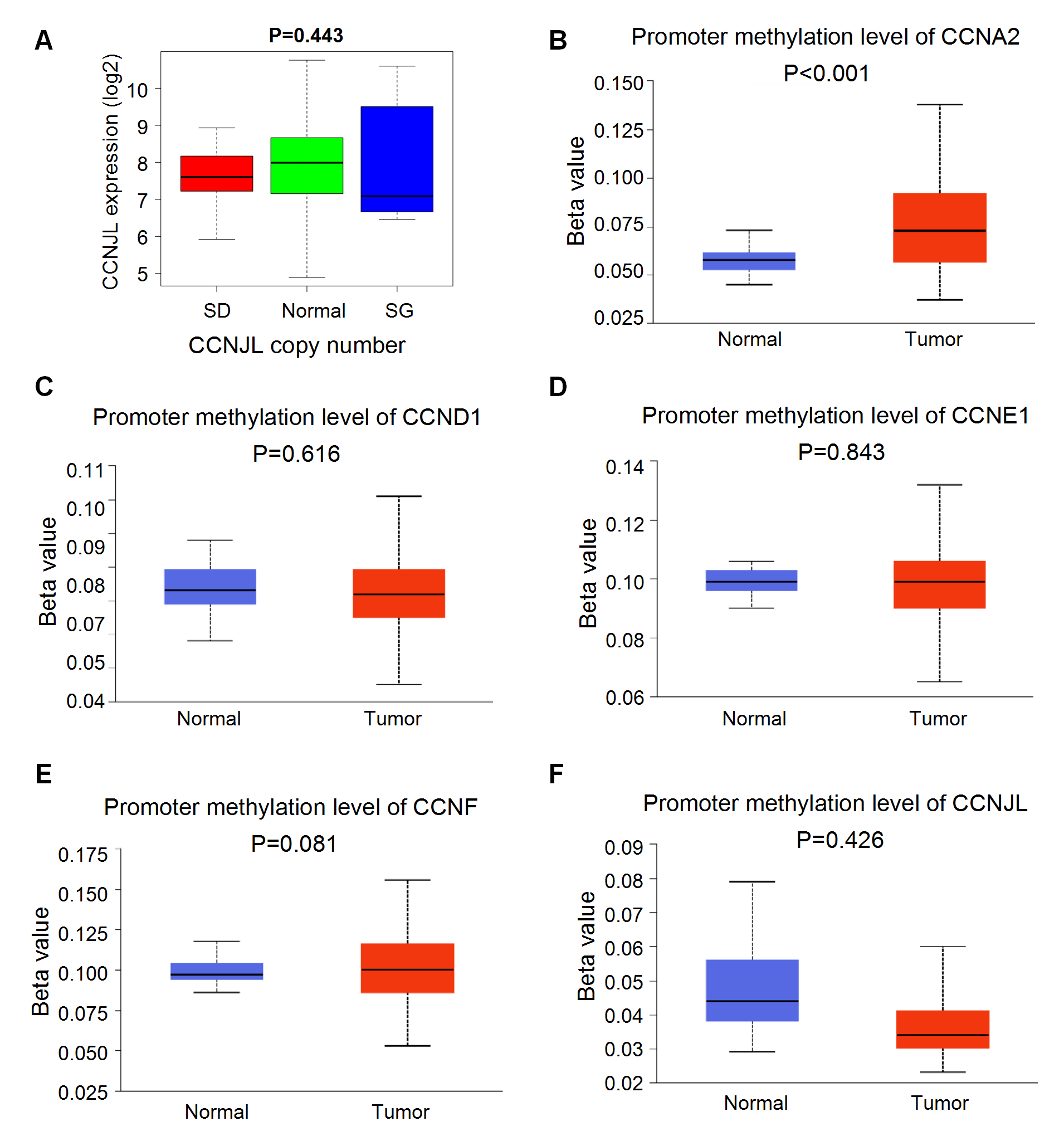


**Supplementary Figure 1.** The copy number variation and DNA methylation of the cyclin DEGs without statistical significance in COAD patients from TCGA. **(A)** The correlation between the copy number and gene expression of CCNJL. SD, single deletion; SG, single gain. **(B-F)** The methylation levels of CCNA2, CCND1, CCNE1, CCNF, and CCNJL in normal and tumor tissues (CCNA2 showed significant difference but its methylation levels were positively associated with expression).


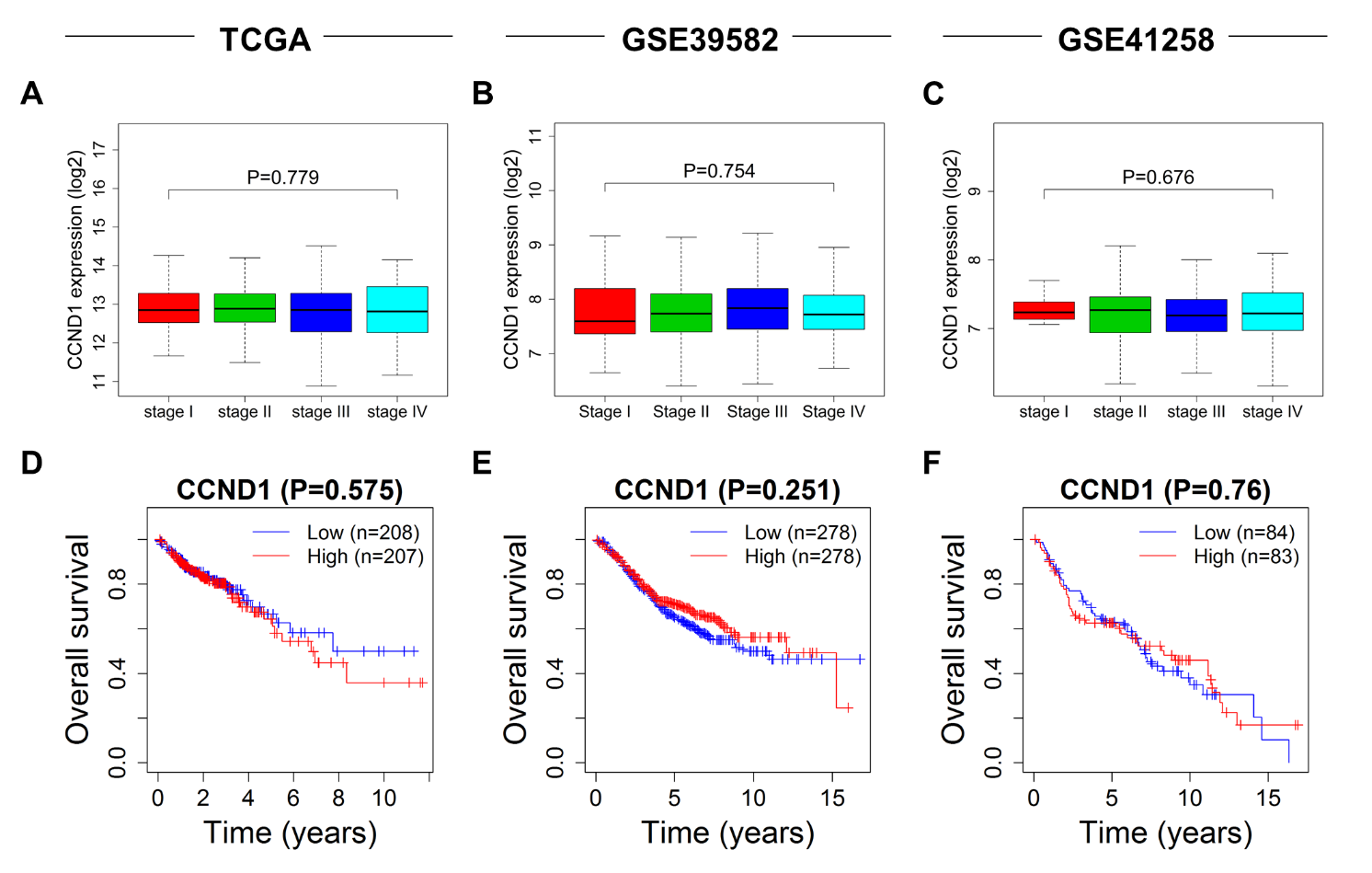


**Supplementary Figure 2.** Gene expression distribution in different TNM stages and survival curves of CCND1 in colon cancer from the three datasets. **(A-C)** The gene expression of CCND1 was not associated with patient TNM stage. **(D-F)** The gene expression of CCND1 was not associated with overall survival.


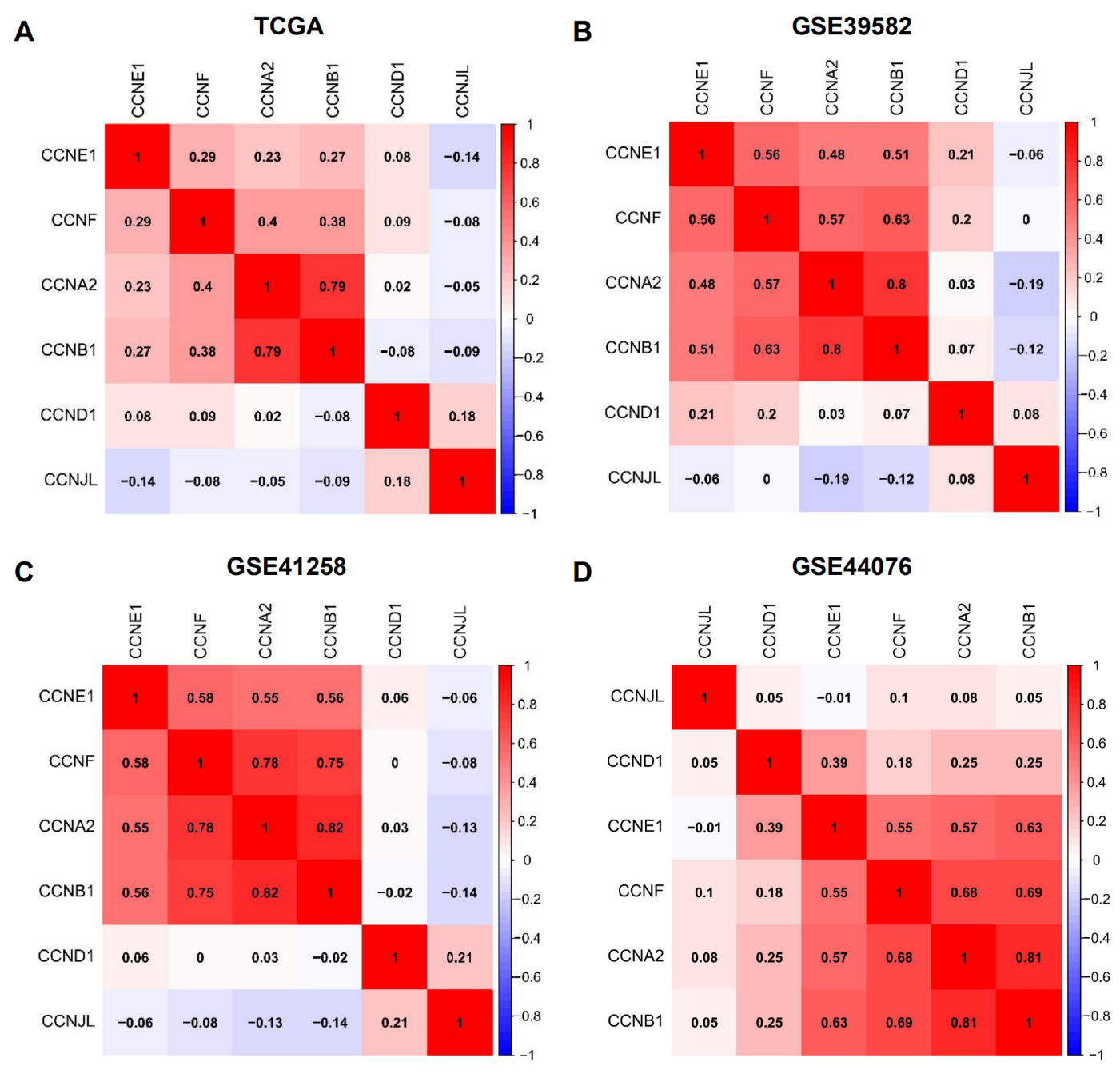


**Supplementary Figure 3.** Pearson correlation coefficients of the six cyclin DEGs in patients with colon cancer from the four datasets.

## Supplementary Tables

**Supplementary Table 1.** Differential expression analysis of cyclin family genes in TCGA

| Gene | logFC | logCPM | P-value | FDR |
| --- | --- | --- | --- | --- |
| CNTD2/CCNP | 4.869 | 2.506 | 0.000 | 0.000 |
| CCNO | 2.986 | 3.417 | 0.000 | 0.000 |
| CCND1 | 1.703 | 7.873 | 0.000 | 0.000 |
| CCNE1 | 1.597 | 3.763 | 0.000 | 0.000 |
| CCNB1 | 1.551 | 6.505 | 0.000 | 0.000 |
| CCNF | 1.452 | 5.125 | 0.000 | 0.000 |
| CCNI2 | 1.218 | 3.979 | 0.000 | 0.000 |
| CCNA2 | 1.175 | 5.723 | 0.000 | 0.000 |
| CCNB2 | 1.043 | 5.503 | 0.000 | 0.000 |
| CCNL2 | 0.968 | 6.492 | 0.000 | 0.000 |
| CCNJ | 0.848 | 3.790 | 0.000 | 0.000 |
| CCNQ | 0.714 | 4.972 | 0.000 | 0.000 |
| CCNE2 | 0.705 | 2.515 | 0.000 | 0.000 |
| CCND2 | 0.695 | 7.708 | 0.000 | 0.000 |
| CCNB3 | 0.471 | -1.082 | 0.035 | 0.049 |
| CCNC | 0.394 | 6.025 | 0.000 | 0.001 |
| CCNH | 0.381 | 4.709 | 0.000 | 0.000 |
| CCNT1 | 0.305 | 5.268 | 0.001 | 0.001 |
| CCNL1 | 0.267 | 5.977 | 0.005 | 0.008 |
| CCNYL2 | 0.138 | -3.298 | 0.844 | 0.868 |
| CCNY | -0.072 | 6.346 | 0.220 | 0.264 |
| CCNI | -0.093 | 8.200 | 0.215 | 0.258 |
| CCNT2 | -0.125 | 5.030 | 0.117 | 0.148 |
| CCNG1 | -0.207 | 6.923 | 0.054 | 0.072 |
| CCNK | -0.268 | 3.633 | 0.002 | 0.003 |
| CCND3 | -0.338 | 6.146 | 0.000 | 0.001 |
| CCNG2 | -0.996 | 5.189 | 0.000 | 0.000 |
| CCNYL1 | -1.411 | 4.665 | 0.000 | 0.000 |
| CCNJL | -1.703 | 3.540 | 0.000 | 0.000 |
| CCNA1 | -1.902 | -2.828 | 0.000 | 0.000 |

FC, fold change; CPM, counts per million; FDR, false discovery rate.

**Supplementary Table 2.** Differential expression analysis of cyclin family genes in GSE39582

| Gene | logFC | Average expression | P-value | Adjusted P-value al |
| --- | --- | --- | --- | --- |
| CCNB1 | 1.575 | 8.060 | 0.000 | 0.000 |
| CCND1 | 1.549 | 7.762 | 0.000 | 0.000 |
| CCNA2 | 1.160 | 7.341 | 0.000 | 0.000 |
| CCNO | 1.001 | 5.687 | 0.000 | 0.000 |
| CCNF | 0.871 | 5.881 | 0.000 | 0.000 |
| CCNE1 | 0.530 | 5.350 | 0.000 | 0.000 |
| CCNJ | 0.343 | 5.065 | 0.000 | 0.000 |
| CCNH | 0.329 | 8.353 | 0.003 | 0.007 |
| CCNI | 0.321 | 8.923 | 0.000 | 0.000 |
| CCNB2 | 0.283 | 5.802 | 0.000 | 0.000 |
| CCNT1 | 0.280 | 7.112 | 0.001 | 0.002 |
| CCND2 | 0.191 | 6.918 | 0.340 | 0.427 |
| CCNY | 0.043 | 6.757 | 0.415 | 0.504 |
| CCNC | 0.037 | 6.362 | 0.557 | 0.639 |
| CCNE2 | -0.017 | 5.598 | 0.930 | 0.949 |
| CCNYL2 | -0.024 | 3.195 | 0.637 | 0.710 |
| CCNG1 | -0.063 | 10.232 | 0.621 | 0.696 |
| CCNL2 | -0.068 | 7.486 | 0.540 | 0.623 |
| CCNB3 | -0.111 | 3.961 | 0.015 | 0.029 |
| CCNK | -0.150 | 6.359 | 0.014 | 0.027 |
| CCNA1 | -0.206 | 2.843 | 0.000 | 0.000 |
| CNTD2/CCNP | -0.280 | 6.585 | 0.000 | 0.000 |
| CCNL1 | -0.300 | 6.331 | 0.008 | 0.017 |
| CCND3 | -0.350 | 6.712 | 0.000 | 0.000 |
| CCNT2 | -0.404 | 5.333 | 0.000 | 0.000 |
| CCNJL | -0.744 | 5.559 | 0.000 | 0.000 |
| CCNG2 | -0.902 | 6.602 | 0.000 | 0.000 |
| CCNYL1 | -1.876 | 6.600 | 0.000 | 0.000 |

FC, fold change.

**Supplementary Table 3.** Differential expression analysis of cyclin family genes in GSE41258

| Gene | logFC | Average expression | P-value | Adjusted P-value al |
| --- | --- | --- | --- | --- |
| CCND1 | 1.151 | 6.927 | 0.000 | 0.000 |
| CCNB1 | 0.963 | 7.130 | 0.000 | 0.000 |
| CCNO | 0.887 | 5.981 | 0.000 | 0.000 |
| CCNF | 0.709 | 5.178 | 0.000 | 0.000 |
| CCNE1 | 0.706 | 6.260 | 0.000 | 0.000 |
| CCNB2 | 0.658 | 7.566 | 0.000 | 0.000 |
| CCNA2 | 0.657 | 6.400 | 0.000 | 0.000 |
| CCNJ | 0.524 | 4.801 | 0.000 | 0.000 |
| CCNE2 | 0.431 | 4.788 | 0.000 | 0.000 |
| CCNL2 | 0.310 | 6.472 | 0.000 | 0.000 |
| CCNC | 0.155 | 7.888 | 0.005 | 0.009 |
| CCNH | 0.084 | 6.987 | 0.101 | 0.139 |
| CCNI | 0.079 | 9.499 | 0.099 | 0.136 |
| CNTD2/CCNP | 0.022 | 6.746 | 0.639 | 0.698 |
| CCND2 | 0.015 | 7.861 | 0.907 | 0.925 |
| CCNT1 | -0.009 | 6.038 | 0.881 | 0.905 |
| CCNK | -0.028 | 3.821 | 0.563 | 0.629 |
| CCNA1 | -0.060 | 4.966 | 0.269 | 0.333 |
| CCNT2 | -0.063 | 5.689 | 0.113 | 0.154 |
| CCNL1 | -0.075 | 8.004 | 0.135 | 0.180 |
| CCND3 | -0.229 | 7.565 | 0.000 | 0.000 |
| CCNG1 | -0.373 | 7.982 | 0.000 | 0.000 |
| CCNG2 | -0.495 | 6.393 | 0.000 | 0.000 |
| CCNJL | -0.512 | 5.193 | 0.000 | 0.000 |

FC, fold change.

**Supplementary Table 4.** Differential expression analysis of cyclin family genes in GSE44076

| Gene | logFC | Average expression | P-value | Adjusted P-value al |
| --- | --- | --- | --- | --- |
| CCNB1 | 1.849 | 7.449 | 0.000 | 0.000 |
| CCND1 | 1.626 | 6.058 | 0.000 | 0.000 |
| CCNA2 | 1.412 | 6.708 | 0.000 | 0.000 |
| CCNF | 0.998 | 4.347 | 0.000 | 0.000 |
| CCNE1 | 0.930 | 4.045 | 0.000 | 0.000 |
| CCNB2 | 0.908 | 7.254 | 0.000 | 0.000 |
| CCNJ | 0.618 | 3.709 | 0.000 | 0.000 |
| CCNE2 | 0.606 | 4.601 | 0.000 | 0.000 |
| CCND2 | 0.516 | 7.445 | 0.000 | 0.000 |
| CCNC | 0.485 | 6.752 | 0.000 | 0.000 |
| CCNI2 | 0.471 | 3.069 | 0.000 | 0.000 |
| CCNO | 0.444 | 2.492 | 0.000 | 0.000 |
| CCNH | 0.405 | 7.198 | 0.000 | 0.000 |
| CCNT1 | 0.246 | 5.873 | 0.000 | 0.000 |
| CNTD2/CCNP | 0.234 | 2.605 | 0.000 | 0.000 |
| CCNG1 | 0.181 | 7.225 | 0.022 | 0.032 |
| CCNY | 0.100 | 5.886 | 0.001 | 0.002 |
| CCNI | 0.087 | 10.007 | 0.100 | 0.130 |
| CCNT2 | 0.006 | 2.848 | 0.850 | 0.875 |
| CCNB3 | -0.011 | 2.420 | 0.706 | 0.748 |
| CCNL2 | -0.020 | 5.149 | 0.687 | 0.731 |
| CCNK | -0.033 | 7.810 | 0.484 | 0.541 |
| CCNA1 | -0.080 | 2.364 | 0.004 | 0.007 |
| CCND3 | -0.238 | 4.577 | 0.000 | 0.000 |
| CCNL1 | -0.340 | 6.441 | 0.000 | 0.000 |
| CCNG2 | -0.487 | 4.398 | 0.000 | 0.000 |
| CCNJL | -0.560 | 3.658 | 0.000 | 0.000 |
| CCNYL1 | -1.145 | 6.591 | 0.000 | 0.000 |

FC, fold change.

**Supplementary Table 5.** Clinical information of the immunohistochemical images from the Human Protein Atlas

| Gene | Tissue type | Patient ID | Age | Gender | Staining |
| --- | --- | --- | --- | --- | --- |
| CCNA2 | Normal | 2040 | 65 | Female | Not detected |
|  | COAD | 4721 | 84 | Female | Medium |
| CCNB1 | Normal | 1958 | 84 | Female | Not detected |
|  | COAD | 1390 | 53 | Female | High |
| CCNE1 | Normal | 1423 | 56 | Female | Not detected |
|  | COAD | 2151 | 75 | Female | Low |
| CCNF | Normal | 2040 | 65 | Female | Not detected |
|  | COAD | 1898 | 71 | Male | Low |
